# Reviewed article: Chagas MEV, Fernandes GR, Fernandes DH, Dode AD, Aguilar GT, Linhares TS, Costa MB, Caires HT, Cabral FC, Constant HMRM, Moreira TC. Specialized medical care in primary care using telemedicine in Northeast Brazil: a descriptive study, Rio Grande do Norte, Brazil, 2022-2023. Epidemiol. Serv. Saúde. 2024;34:e20240256. 10.1590/S2237-96222024v34e20240256.en

**DOI:** 10.1590/S2237-96222025v34e20240256.a

**Published:** 2025-03-14

**Authors:** Mariana Aparecida Brozoski

**Affiliations:** 1 Universidade de São Paulo, São Paulo, SP, Brazil Universidade de São Paulo São Paulo SP Brazil

## Peer review A

## Questionnaire

Does the manuscript contain new and significant information to justify publication? Yes

Does the Abstract (Summary) clearly and accurately describe the content of the article? Yes

Is the problem significant and concisely stated? Yes

Are the methods described comprehensively? Yes

Are the interpretations and conclusions justified by the results? Yes

Is adequate reference made to other work in the field? Yes

## Manuscript Structure

Length of article is: Adequate

Number of tables is: Too many

Number of figures is: Adequate

**Please state any conflict(s) of interest that you have in relation to the review of this paper (state “none” if this is not applicable).**

None

**Interest:** Good

**Quality:** Good

**Originality:** Good

**Overall:** Good

**Recommendation:** Accept

## Comments

Artigo bem delineado considero adequado para publicação. A sugestão seria continuar o a pesquisa como vocês mesmo sugerem com um acompanhamento maior indo maior robustez para o uso da telemedicina com especialistas como uma alternativa para areas com escassez profissional.

